# Comparative Host Feeding Patterns of the Asian Tiger Mosquito, *Aedes albopictus*, in Urban and Suburban Northeastern USA and Implications for Disease Transmission

**DOI:** 10.1371/journal.pntd.0003037

**Published:** 2014-08-07

**Authors:** Ary Faraji, Andrea Egizi, Dina M. Fonseca, Isik Unlu, Taryn Crepeau, Sean P. Healy, Randy Gaugler

**Affiliations:** 1 Center for Vector Biology, Department of Entomology, Rutgers University, New Brunswick, New Jersey, United States of America; 2 Mercer County Mosquito Control, West Trenton, New Jersey, United States of America; 3 Monmouth County Mosquito Extermination Commission, Eatontown, New Jersey, United States of America; North Carolina State University, United States of America

## Abstract

**Background:**

*Aedes albopictus* is an invasive species which continues expanding its geographic range and involvement in mosquito-borne diseases such as chikungunya and dengue. Host selection patterns by invasive mosquitoes are critically important because they increase endemic disease transmission and drive outbreaks of exotic pathogens. Traditionally, *Ae. albopictus* has been characterized as an opportunistic feeder, primarily feeding on mammalian hosts but occasionally acquiring blood from avian sources as well. However, limited information is available on their feeding patterns in temperate regions of their expanded range. Because of the increasing expansion and abundance of *Ae. albopictus* and the escalating diagnoses of exotic pathogens in travelers returning from endemic areas, we investigated the host feeding patterns of this species in newly invaded areas to further shed light on its role in disease ecology and assess the public health threat of an exotic arbovirus outbreak.

**Methodology/Principal Findings:**

We identified the vertebrate source of 165 blood meals in *Ae. albopictus* collected between 2008 and 2011 from urban and suburban areas in northeastern USA. We used a network of Biogents Sentinel traps, which enhance *Ae. albopictus* capture counts, to conduct our collections of blooded mosquitoes. We also analyzed blooded *Culex* mosquitoes collected alongside *Ae. albopictus* in order to examine the composition of the community of blood sources. We found no evidence of bias since as expected *Culex* blood meals were predominantly from birds (n = 149, 93.7%) with only a small proportion feeding on mammals (n = 10, 6.3%). In contrast, *Aedes albopictus* fed exclusively on mammalian hosts with over 90% of their blood meals derived from humans (n = 96, 58.2%) and domesticated pets (n = 38, 23.0% cats; and n = 24, 14.6% dogs). *Aedes albopictus* fed from humans significantly more often in suburban than in urban areas (χ^2^, p = 0.004) and cat-derived blood meals were greater in urban habitats (χ^2^, p = 0.022). Avian-derived blood meals were not detected in any of the *Ae. albopictus* tested.

**Conclusions/Significance:**

The high mammalian affinity of *Ae. albopictus* suggests that this species will be an efficient vector of mammal- and human-driven zoonoses such as La Crosse, dengue, and chikungunya viruses. The lack of blood meals obtained from birds by *Ae. albopictus* suggest that this species may have limited exposure to endemic avian zoonoses such as St. Louis encephalitis and West Nile virus, which already circulate in the USA. However, growing populations of *Ae. albopictus* in major metropolitan urban and suburban centers, make a large autochthonous outbreak of an arbovirus such as chikungunya or dengue viruses a clear and present danger. Given the difficulties of *Ae. albopictus* suppression, we recommend that public health practitioners and policy makers install proactive measures for the imminent mitigation of an exotic pathogen outbreak.

## Author Summary

*Aedes albopictus* is one of the most invasive and aggressive disease vectors in the world. The range of this species is currently still expanding, particularly into highly dense human population centers in temperate areas in the USA and Europe, raising the public health threat of emerging and re-emerging diseases such as chikungunya and dengue. The prominence of *Ae. albopictus* as a major vector was exposed during the global pandemic of chikungunya virus, primarily because of a virus adaptation which enhanced the transmission efficiency by this mosquito species and also because of the first locally-transmitted cases of chikungunya virus in temperate Europe. Blood feeding patterns by mosquitoes are a critical component of virus proliferation and determine the degree and intensity of disease epidemics, particularly in newly invaded areas. We examined the blood meal sources of invasive *Ae. albopictus* in the northernmost boundary of their range in temperate North America and found that the species fed exclusively on mammalian hosts, with over 90% of their blood meals derived from humans and their associated pets (cats and dogs). The high mammalian affinity of *Ae. albopictus* suggests that this species may be an efficient vector of mammal-driven zoonoses and human-driven anthroponoses such as dengue and chikungunya viruses in this region.

## Introduction

Understanding the blood feeding patterns of mosquitoes is of paramount importance in determining their vector status in the maintenance and epidemic transmission of arboviruses. Blood feeding patterns of mosquito vectors provide insight into the ecological transmission cycles of pathogens and lead to more efficient disease and vector control measures for the benefit of animal and human health. For invasive mosquitoes with expanding geographic ranges, such as *Aedes albopictus* (Skuse), the specific blood-hosts impact endemic diseases and can lead to the epidemic transmission of exotic pathogens.

The Asian tiger mosquito, *Ae. albopictus*, has dispersed extensively from its native tropical range in Southeast Asia and is now found on every continent except Antarctica [1], [2]. The last decade has seen a dramatic expansion of *Ae. albopictus* into temperate regions of Europe and North America [3]–[5]. In many parts of its expanded range, this species is implicated as a significant vector of emerging and re-emerging arboviruses such as dengue (DENV) and chikungunya (CHIKV).

Although historically not an important vector of CHIKV, *Ae. albopictus* has become the principal driver of recent epidemics in Asia and islands in the Indian Ocean because of a mutation in the virus envelope protein enhanced transmission efficiency by this species [6], [7]. Autochthonous transmission of CHIKV has also been recorded in temperate regions of Italy and France [8], [9] where invasive *Ae. albopictus* have become abundant [3]. *Aedes albopictus* was also the sole vector in local epidemics of dengue in Hawai'i and other regions [10], [11] and is a competent laboratory vector for at least 22 arboviruses [12]. Due to the widespread and increasing distribution of *Ae. albopictus* in temperate regions and the escalating diagnoses of exotic pathogens in travelers returning from endemic or epidemic areas [13], [14], the risk of an outbreak in a new area is no longer hypothetical. Furthermore, because this species thrives in artificial containers found in close association with human peridomestic environments, it is essential to fully investigate the host feeding patterns of *Ae. albopictus* in order to completely understand its role in disease ecology and public health significance.

Surprisingly, given the vector potential and medical importance of *Ae. albopictus*, few studies have been conducted to investigate the host feeding patterns of this species in its native and expanding geographic range. This is likely because adult *Ae. albopictus* are a difficult species to collect efficiently in traps, and blood fed specimens are especially rare. From the few studies that have been conducted, the precise host feeding preferences of *Ae. albopictus* seem to vary considerably (Table 1). The species has been generally reported to feed on a wide range of mammals including humans, but will also feed on avian hosts at various proportions, and has even been incriminated to feed on amphibians and reptiles [15]–[34]. It has thus been considered an opportunistic feeder and a classic bridge vector candidate between zoonotic arboviruses and humans. However, caution should be taken in labeling *Ae. albopictus* as an efficient bridge vector because the large variation in the feeding plasticity of this species questions the exact role that it may play as an enzootic or epidemic vector of arboviruses. For example, in its native tropical range, *Ae. albopictus* feeds exclusively on humans in Indonesia [35], whereas in Singapore it feeds on humans, oxen, and dogs [15]. Additionally, studies conducted in Thailand [36] have reported that *Ae. albopictus* feed on humans, swine, buffalo, dogs, and chickens, while more recent investigations [26] report that *Ae. albopictus* feeds only on humans, with a few (<6%) double-host blood meals between humans and swine/cat/dog. In temperate Japan, *Ae. albopictus* primarily feed on mammals, with a high propensity for humans, but also on birds and amphibians/reptiles [29], [30] (Table 1).

**Table 1:** Literature review of the host feeding preferences of *Aedes albopictus* in its native and invasive geographic range.

Geographic Range	Location	Habitat Type	Trap Type	Bloodmeal Assay	No. Identified	Host Class %				Reference
						Mammal (Human)	Avian	Herptile	>1 Host*	
Native	Japan	Suburban/Urban	CDC, SN	PCR	114	84.2 (68.5)	6.1	3.5	6.1	Sawabe *et al.* 2010
Native	Japan	Rural	BGS, CDC, SN	PCR	13	100 (30.8)				Kim *et al.* 2009
Native	Thailand	Rural	ASP	ELISA	105	100 (94.3)			5.7	Ponlawat & Harrington 2005
Native	China	Rural	ASP	ELISA	48	75.0 (63.9)	25.0			Almeida *et al.* 2005
Native	India	Cattle shed	ASP	Precipitin	40	100 (ND)				Tandon & Ray 2000
		Suburban			162	100 (100)				
		Urban			362	81.8 (98.7)	10.5		7.7	
Native	Singapore	Rural	ASP, UTN	Precipitin	37	100 (91.9)				Colless 1959
Old World Invasive	Spain	Urban	BGS	PCR	30	100 (100)				Munoz *et al.* 2011
Old World Invasive	Cameroon	Rural	SN	ELISA	170	96.3 (100)		0.6	3.1	Kamgang *et al.* 2012
Old World Invasive	Italy	Rural	ST	ELISA	60	65.0 (30.8)	5.0		30.0	Valerio *et al.* 2010
		Urban			243	92.2 (91.1)	0.8		7.0	
New World Invasive	USA	Zoo	ASP, GT	PCR	5	40.0 (ND)	60.0			Tuten *et al.* 2012
New World Invasive	USA	Urban	GT	PCR	9	100 (44.4)				Dennett *et al.* 2007
New World Invasive	USA	Suburban	ASP	ELISA, PCR	1,094	83.1 (24.1)	7.5	3.4	5.1	Richards *et al.* 2006
New World Invasive	USA	Rural/Suburban	CDC, HL, GT	ELISA	22	81.8 (ND)	4.6		13.6	Gingrich & Williams 2005
New World Invasive	Brazil	Urban	ASP, SN	Precipitin	177	97.7 (68.2)	2.3			Gomes *et al.* 2003
New World Invasive	USA	Rural	ASP	ELISA	93	93.6 (8.1)	1.1	5.4		Niebylski *et al.* 1994
		Urban			152	98.7 (2.0)	1.3			
New World Invasive	USA	Tire dump	ASP, CDC, HL, SN	ELISA	139	79.1 (8.2)	20.9			Savage *et al.* 1993
New World Invasive	USA	Rural	ASP	Precipitin	1,075	93.7 (19.4)	5.8		0.6	Tempelis *et al.* 1970
New World Invasive	USA	Rural	ASP	Precipitin	41	27.0 (ND)	73.0			Hess *et al.* 1968
		Suburban			14	93.0 (7.1)	7.0			

All collections were conducted under field settings using various trapping methods as indicated. Table excludes laboratory or field host-choice experiments.

*Includes specimens with mixed blood meals from more than one vertebrate hosts.

ELISA = enzyme-linked immunosorbent assay; ND = non detected; PCR = polymerase chain reaction. ASP = aspirator; BGS = Biogents Sentinel trap; CDCLT = Centers for Disease Control light trap; GT = gravid trap; HL = human landing; SN = sweep net; ST = sticky trap; UTN = unbaited trap net.

In temperate locations of the expanding range of *Ae. albopictus*, the host preference of this species is also variable. Studies conducted at a tire dump in Missouri, USA, reported that *Ae. albopictus* will feed on birds (17%) but prefer mammals (64%), with 8.2% of those mammalian feedings obtained from humans [19]. A follow up study conducted in other tire yards and surrounding vegetation of rural and urban habitats in Missouri, Florida, Indiana, Illinois, and Louisiana, USA, concluded that *Ae. albopictus* showed a strong preference for mammals (>94%), with up to 8% human-derived blood meals, while also detecting avian (1%) and reptilian (5%) blood meals [20]. An additional study in suburban landscapes of North Carolina, USA, reported that *Ae. albopictus* feeds predominately on mammalian hosts (83%), but also on birds (7%), amphibians (2%), and reptiles (2%) [27]. In Europe, Italian populations of *Ae. albopictus* rarely feed on birds in urban settings, while 99% of specimens have been reported to feed on mammals, with 90% of those mammalian blood meals being derived from humans [31]. The same investigators report that in suburban settings of Italy, 7% of *Ae. albopictus* had fed on avian species, while the vast majority of the blood meals were mammalian-derived (95%), with 43% containing human blood [31]. Finally, in urban zones of Spain, *Ae. albopictus* obtained blood meals exclusively from humans (100%) [32] (Table 1).

Although it is apparent that *Ae. albopictus* feeds predominantly on mammals, the degree of mammalophagic or anthropophagic host feeding preferences of this species appear location specific. Because of the rapidly expanding range of *Ae. albopictus*, its abundance in metropolitan centers, and its close association with humans in peridomestic habits, combined with the emergence and resurgence of exotic pathogens for which *Ae. albopictus* is a capable vector, it is clear that assessing its host feeding preferences in newly invaded areas is critical to elucidate disease transmission cycles and develop strategies to reduce the local risk of an exotic arbovirus outbreak. However, the collection of *Aedes* (*Stegomyia*) spp., such as *Ae. albopictus*, has been difficult because standard vector surveillance traps are generally placed 1.5 m above the ground, are operated overnight, and utilize light as an attractant [37]. Since *Ae. albopictus* is diurnal and not attracted to light, host-seeks near the ground surface, and utilizes visual, in addition to olfactory cues for host location [18], [21], [38] these traps are not an effective way to collect this species. Consequently, most blood meal analyses to date were performed on specimens collected from areas where their densities are very high, such as tire yards and tire dumps (Table 1). The creation of newly developed vector surveillance traps, such as the Biogents Sentinel (BGS) trap, have only recently allowed the collection of large number of *Ae. albopictus* specimens from typical urban and suburban areas for ecological studies [39]. These traps simulate convection currents created by human body heat, utilize lures which mimic human odors, are operated during the day, placed at the ground level, and utilize contrasting black and white markings that provide additional visual cues that may be attractive to *Ae. albopictus* [37]–[41].

We investigated the host feeding patterns of *Ae. albopictus* in temperate North America, near the northernmost boundary of established populations in the eastern United States [4], [5]. We used an extensive network of BGS traps, which enhance *Ae. albopictus* capture counts, to conduct a multi-year collection of blooded mosquitoes (2008–2011) in urban and suburban sites as part of a larger area-wide project aimed at managing the Asian tiger mosquito [42], [43]. Additionally, we assayed blood meals from *Culex* mosquitoes collected in the same traps, locations, and dates as *Ae. albopictus* to determine the diversity of different blood meal sources obtained from the two vectors. We discuss the implications of our results on established and expanding populations of *Ae. albopictus* and the imminent outbreaks of exotic diseases such as chikungunya or dengue fevers in North America.

## Materials and Methods

### Statement of Ethics

All studies were conducted within the jurisdictions of the authors' respective governance domain by professional mosquito control personnel. All entomological surveys and collections made on private lands or in private residences were conducted after acquisition of oral or written consent from residents. No specific permits were required for the mosquito collections. These studies did not involve endangered or protected species.

### Study Area

All collections were conducted within two counties (Mercer and Monmouth) located in central New Jersey, USA. Mercer County (40°13′N, 74°44′W) is highly urban, with 364,883 residents [44] and a population density of 630.2 inhabitants per square kilometer. Mercer County and the low-income City of Trenton, where the studies were conducted, have a population density of 4,286.5/km^2^ (USCB 2009a). The City of Trenton contains typical dense inner city housing, often built as adjoining row homes or duplexes [45]. Monmouth County (40°44′N, 74°17′W) is defined as primarily suburban and is located in east-central New Jersey with a population of 630,380 [46]. The boroughs on the Raritan Bayshore, within Monmouth County, where the studies were conducted, have an average population of 1,907.4/km^2^ [46]. The Raritan Bayshore primarily contains middle income coastal suburban homes which are often interspersed with forest and green space remnants [42]. Within each county, three predefined ∼1,000-parcel sites (a parcel is a combination of a house and its associated yard space), ranging in area from 1 km^2^ (Mercer) to 2 km^2^ (Monmouth) were chosen for our investigations. Although individual parcel sizes within the study sites in Mercer County were smaller (199.5±18.3 m^2^) than those in Monmouth County (571.1±31.2 m^2^), the number of residents within Mercer sites (19,494) were larger than within Monmouth sites (12,743). Every site, within each county, was previously selected to contain similar socioeconomic parameters, geography, human population density, and mosquito abundance. For a detailed description about site selection and the parameters of each individual site, please refer to [42], [43].

### Mosquito Surveillance

Mosquitoes were sampled on a weekly basis during 2008–2011 using a network of Biogents Sentinel (BGS) traps (Biogents AG, Regensburg, Germany). Specific details of surveillance protocols are outlined elsewhere [40]–[43], [47]; but briefly, trap locations were chosen by overlaying a grid of specific distance intervals. We used a 175–200 m distance between BGS traps for each site in Mercer County and 200–400 m distances in Monmouth County because of the larger site areas and limiting number of traps in inventory. These distances were based on current knowledge of *Ae. albopictus* flight range [21] and the available resources within each county. A total of 36 to 51 BGS traps, depending on the year, were deployed weekly in Mercer County, while 55 to 57 traps were deployed in Monmouth County. Each BGS trap was placed in residential backyards (near vegetation or shade) of each parcel selected, and was operated for 24 hours prior to collection. Each week, traps were placed in the same location within the backyards. The BGS trap was used with a solid BG-lure (Biogents AG, Regensburg, Germany) containing ammonia, lactic acid and fatty acids, components known to be attractive to *Ae. albopictus* [37]. Although the BGS trap was designed to capture host seeking (unfed) *Aedes* (*Stegomyia*) mosquitoes [39], the trap also captures other species such as *Culex* mosquitoes [37], [42] in addition to occasionally collecting female mosquitoes in varying gonotrophic stages (unengorged, blood fed, black blooded, and gravid). An unengorged or unfed mosquito does not contain visible evidence of blood in the abdomen, while a blood fed mosquito displays a distended abdomen with reddish blood clearly visible. A black blooded specimen has digested most of the blood meal and retains only a small portion of dark red or black blood visible near the ventral anterior of the abdomen, corresponding with Sella stage VI [48]. Gravid specimens have completely digested blood meals and contain visible eggs ready for oviposition.

Collections were placed on dry ice immediately and transported to the laboratory for identification and pooling. Species identification, enumeration, and gonotrophic stage determination was conducted under a dissecting microscope using a chill table to maintain a cold chain. Specimens were stored at −80°C for subsequent blood meal determination.

### Blood Meal Identification from *Ae. albopictus*

Abdomens of blooded *Ae. albopictus* were dissected over a chill table and then extracted using a Qiagen DNeasy Blood and Tissue Kit (Qiagen Sciences, Germantown, MD, USA). Specimens with very small blood remnants or those deemed poorly preserved (desiccated), were not utilized for DNA extraction because those samples rarely yield useful data [49]. To avoid contamination, forceps were flamed between extractions. To save time and reagents, we used a strategy that allows rapid identification of human-derived blood meals and mixes between human and non-human mammals [49]. This technique identifies human-derived blood meals based on the size of the PCR product on a gel without the need for extensive sequencing, thus drastically reducing costs. A mix between human and non-human blood is detected as two bands, and only the non-human band must be excised from the gel and purified with a QIAquick Gel Extraction Kit (Qiagen, Valencia, CA, USA) prior to sequencing [49]. Samples that did not amplify with the above assay were also tested with previously established primers designed for birds [50], reptiles/amphibians [51], and an additional primer set for mammals [52]. Approximately half of the specimens were tested with all bloodmeal identification methods above to legitimize the use of the rapid-assay [49]. To test for contamination, negative controls were employed in all reactions. The negative controls consisted of the PCR master mix with sterile water. Except for the short human-only band obtained with the Egizi et al. assay [49], and when the non-human band was excised from the agarose gel (see above), all PCR products were cleaned with Exo-Sap-IT (USB Products, Cleveland, OH, USA), cycle-sequenced with the forward primer of each pair, and run on capillary automated sequencers. Sequences were BLASTed in GenBank (http://www.ncbi.nlm.nih.gov/blast/Blast.cgi) to compare with sequences of known species. Only matches of >98% similarity were identified as the source of the blood meal [53].

### Molecular Identification and Blood Meal Analyses of *Culex* Mosquitoes

A large number of blooded *Culex* mosquitoes, consisting primarily of *Culex pipiens pipiens* L. and *Culex restuans* Theobald, were also collected by the BGS traps. Because of the difficulty in accurate morphological identification of field-collected specimens due to age or damage [54]–[56] these specimens are often pooled as *Culex* spp. After using a molecular assay to identify all *Culex* mosquitoes to species [57], we tested blood fed *Culex* specimens from both counties collected in the same traps, locations, and dates as *Ae. albopictus*. *Culex p. pipiens* and *Cx. restuans* were the only *Culex* species collected in the BGS traps, and were assayed from Mercer County during 2009–2011 and from Monmouth County during 2008 and 2011. Blooded *Culex* specimens were extracted as described above for *Ae. albopictus*, amplified with the BM primer pair [58], then cleaned, sequenced, and identified as above. The BM primer pair targets a wide range of species, including mammals, birds, and reptiles, but it inadvertently amplifies in *Ae. albopictus* [49] and therefore cannot be used to identify blood meals in that species.

### Data Analyses

Spatial differences in the proportion of *Ae. albopictus* feeding on selected host species between the counties was compared by using Pearsons χ^2^ analysis for trend. All analyses were performed using IBM SPSS Statistics 21 (IBM, Armonk, NY, USA). Confidence intervals surrounding the estimated proportion of blood meals taken from a given species were calculated using the formula 95% CI = ±1.96×(square root *p* (1−*p*)/n), where *p* = the proportion of blood meals from a given source, and *n* = the total number of blood meals identified [59].

## Results

### Mosquito Surveillance

Our BGS trap surveillance during the active mosquito seasons of 2008–2011 collected 73,828 *Ae. albopictus* females in Mercer and Monmouth Counties (Table S1). A total of 33,392 *Ae. albopictus* were collected in Mercer County, 187 (0.56%) of which were visually determined to contain blood (blood fed or black blooded, hereafter “blooded”); while 40,436 *Ae. albopictus* were collected in Monmouth County, with 219 (0.54%) containing blood. In Mercer County, the number and proportion of blooded *Ae. albopictus* collected during each month was as follows: May (n = 1, 1.25% of monthly total), June (13, 0.82%), July (23, 0.42%), August (70, 0.57%), September (61, 0.57%), and October (19, 0.60%). Blooded *Ae. albopictus* in Monmouth County were collected during May (n = 4, 1.24% of monthly total), June (25, 1.11%), July (65, 0.99%), August (72, 0.45%), September (37, 0.33%), and October (16 (0.56%). We also captured 14,989 *Culex* mosquitoes (*Cx. p. pipiens*, *Cx. restuans*, and *Cx.* spp.) from both counties (Table S2). The BGS trap is highly specific for capturing host seeking *Ae. albopictus* females, as apparent by the nearly 74,000 specimens of this species that were captured versus the 15,000 specimens of *Culex* mosquitoes (Tables S1, S2). Interestingly, BGS traps were also capable of capturing blooded *Ae. albopictus* and *Culex* mosquitoes, as evidenced by the collection of over 406 blooded *Ae. albopictus* and 745 blooded *Culex* (Tables S1, S2).

### Blood Meal Identification from *Ae. albopictus*

Of the 406 blooded *Ae. albopictus* collected, 117 individuals were too desiccated and therefore only 289 specimens were suitable for dissection. Subsequently, the blood meal origin of 165 (57.10%) specimens was successfully determined (Table S1, 2). In Mercer County, 125 were tested for host blood meal origination with a successful identification from 86 (68.80%) specimens (Table 2). In Monmouth County, 164 *Ae. albopictus* were tested, with a successful host determination from 79 (48.17%) of those specimens (Table 2).

**Table 2:** Origin of blood meals obtained from *Aedes albopictus* in urban (Mercer County) and suburban (Monmouth County) habitats during 2008–2011.

	Mercer County (% [95% CI])					Monmouth County (% [95% CI])					Total
Host Species	2008	2009	2010	2011	Subtotal	2008	2009	2010	2011	Subtotal	
Human (*Homo sapiens*)	19 (45.2 [30.2–60.3])	4 (33.3 [6.7–60.0])	2 (13.3 [0.1–30.5])	12 (70.6 [48.9–92.3])	37 (43.0 [32.6–53.5])	23 (59.0 [43.5–74.4])	6 (66.7 [35.9–97.5])	5 (83.3 [53.5–99.0])	15 (60.0 [40.8–79.2])	49 (62.0 [51.3–72.7])	86 (52.1 [44.5–59.7])
Cat (*Felis catus*)	10 (23.8 [10.9–36.7])	5 (41.7 [13.8–69.6])	6 (40.0 [15.2–64.8])	3 (17.6 [0.1–35.8])	24 (27.9 [18.4–37.4])	6 (15.4 [4.1–26.7])			4 (16.0 [1.6–30.4])	10 (12.7 [5.3–20.0])	34 (20.6 [14.4–26.8])
Dog (*Canis familiaris*)	7 (16.7 [5.4–28.0])	3 (25.0 [0.5–49.5])	3 (20.0 [0.1–40.2])	1 (5.9 [0.1–17.1])	14 (16.3 [8.5–24.1])	2 (5.1 [0.1–12.1])	1 (11.1 [0.1–31.6])		2 (8.0 [0.1–18.6])	5 (6.3 [1.0–11.7])	19 (11.5 [6.6–16.4])
Virginia opossum (*Didelphis virginiana*)	2 (4.8 [0.1–11.2])		2 (13.3 [0.1–30.5])		4 (4.7 [0.2–9.1])	1 (2.6 [0.1–7.5])			2 (8.0 [0.1–18.6])	3 (3.8 [0.1–8.0])	7 (4.2 [1.2–7.3])
Gray squirrel (*Sciurus carolinensis*)	2 (4.8 [0.1–11.2])			1 (5.9 [0.1–17.1])	3 (3.5 [0.1–7.4])	2 (5.1 [0.1–12.1])			1 (4.0 [0.1–11.7])	3 (3.8 [0.1–8.0])	6 (3.6 [0.8–6.5])
Cottontail rabbit (*Sylvialagus floridanus*)							2 (22.2 [0.1–49.4])			2 (2.5 [0.1–6.0])	2 (1.2 [0.1–2.9])
White-footed mouse (*Peromyscus leucopus*)								1 (16.7 [0.1–46.5])		1 (1.3 [0.1–3.7])	1 (0.6 [0.1–1.8])
Human+Dog	1 (2.4 [0.1–7.0])		1 (6.7 [0.1–19.3])		2 (2.3 [0.1–5.5])	3 (7.7 [0.1–16.1])				3 (3.8 [0.1–8.0])	5 (3.0 [0.4–5.7])
Human+Cat	1 (2.4 [0.1–7.0])		1 (6.7 [0.1–19.3])		2 (2.3 [0.1–5.5])	1 (2.6 [0.1–7.5])			1 (4.0 [0.1–11.7])	2 (2.5 [0.1–6.0])	4 (2.4 [0.1–4.8])
Human+Deer (*Odocoileus virginianus*)						1 (2.6 [0.1–7.5])				1 (1.3 [0.1–3.7])	1 (0.6 [0.1–1.8])
Total no. identified (%)	42 (84.0)	12 (75.0)	15 (65.2)	17 (47.2)	86 (68.8)	39 (62.9)	9 (45.0)	6 (16.2)	25 (55.6)	79 (48.2)	165 (57.1)
Total no. tested	50	16	23	36	125	62	20	37	45	164	289

Percentages are provided in parentheses followed by ±95% CI.

*Aedes albopictus* fed exclusively on mammalian hosts in Mercer and Monmouth Counties, with over 84% of all identified blood meals stemming from humans (52.12%), cats (20.61%), or dogs (11.52%) (Table 2). Blood meals were also detected from opossums (4.24%), gray squirrels (3.64%), cottontail rabbits (1.21%), and a white-footed mouse (0.61%). A small percentage (6.06%) of double blood meals (from two different host species) were detected in *Ae. albopictus* (4.65% of total in Mercer and 7.60% of total in Monmouth), and all included human blood (human+dog, n = 5; human+cat, n = 4; human+deer, n = 1). The number of *Ae. albopictus* feeding on humans was significantly higher in suburban Monmouth (62%) than in urban Mercer (43%) County locations (χ^2^ = 8.151; df = 1; p = 0.004), but significantly more *Ae. albopictus* fed on cats in Mercer than in Monmouth County (χ^2^ = 5.256; df = 1; p = 0.022). No significant difference was observed in the number of *Ae. albopictus* feeding on dogs between the two counties. No avian-derived blood meals were detected in any of the *Ae. albopictus* specimens tested.

**Figure 1:**
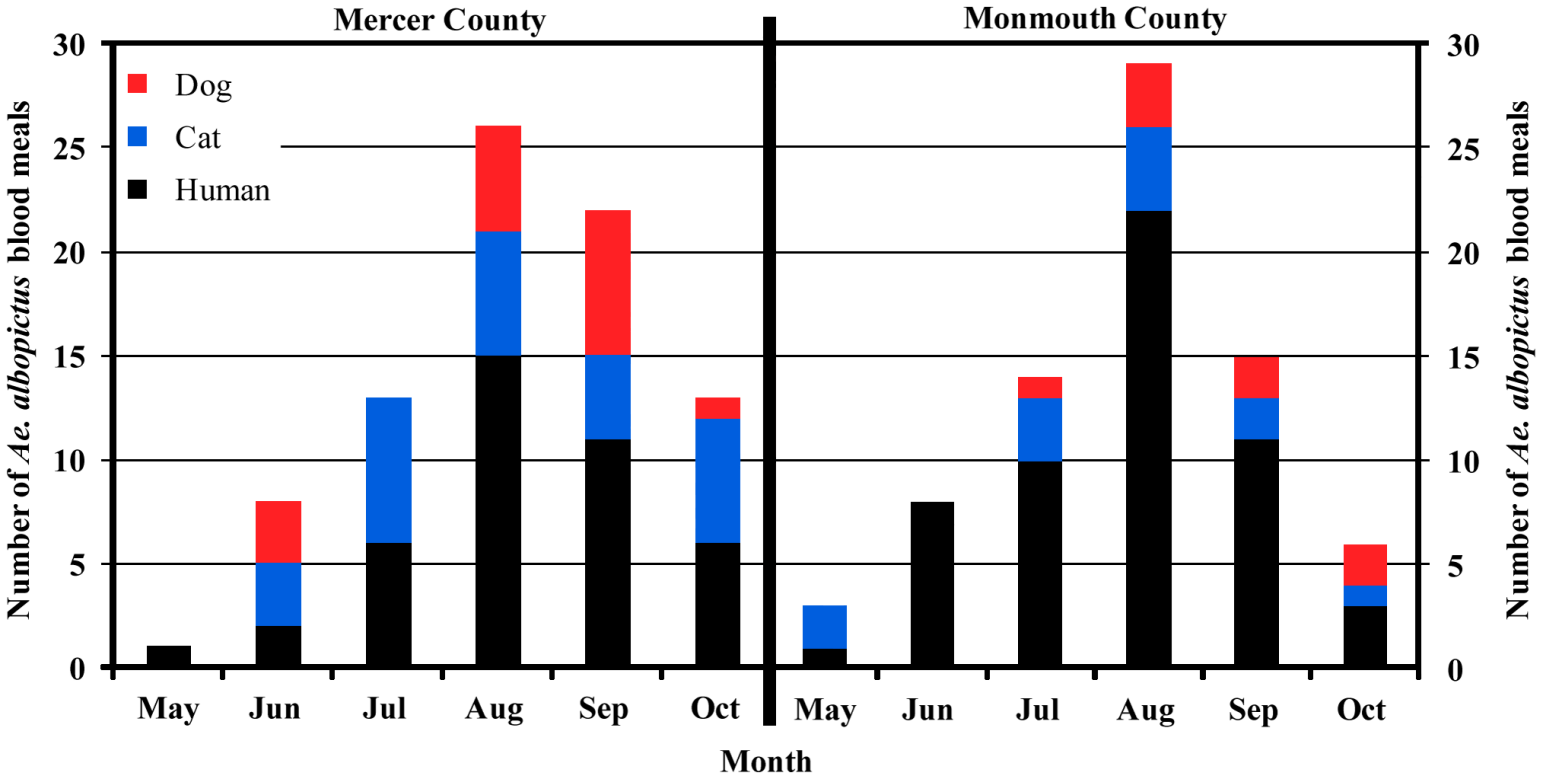
Monthly number of *Aedes albopictus*-derived blood meals from cats, dogs, and humans in urban (Mercer County) and suburban (Monmouth County) habitats of northeastern USA (2008–2011).

Human- and cat-derived blood meals in *Ae. albopictus* were detected every month of our studies, while dog-derived blood meals were absent during May (Figure 1). Only 2.08% of all human-derived blood meals were detected in May, while the vast majority was detected during the month of August (38.54%). Four contiguous months (July, August, September, and October) accounted for over 87% of all blood meal collections (Figure 1).

### Blood Meal Analyses and Molecular Identification of *Culex* Mosquitoes

We collected 745 blooded *Culex* (349 *Cx. p. pipiens*, 181 *Cx. restuans*, 215 *Cx.* spp.) mosquitoes during 2008–2011, and tested a subsample of 198 individuals identified as *Cx. p. pipiens* or *Cx. restuans* for blood meal source determination (Table 3). We selected 198 specimens to approximate the number of blood meals identified from *Ae. albopictus* and chose specimens from the same dates and traps as feasible. We were able to identify the blood meal source of 159 (80.30%) samples. Blooded *Cx. p. pipiens* were collected during April (n = 1, 0.79%), May (19, 15.08%), June (37, 29.37%), July (26 (20.63%), August (19, 15.08%), September (21, 16.67%), and October (3, 2.38%). Blooded *Cx. restuans* were collected during May (n = 10, 30.30%), June (12, 36.36%), July (6, 18.18%), August (2, 6.06%), September (2, 6.06%), and October (1, 3.03%). In Mercer County, specimens were tested from 2009–2011 and resulted in successful host determination from 61 *Cx. p. pipiens* (n = 74, 82.43%) and 7 *Cx. restuans* (n = 7, 100%). In Monmouth County, the blood meal hosts of 65 *Cx. p. pipiens* (n = 80, 81.25%) and 26 *Cx. restuans* (n = 37, 70.27%) were determined from 2008 and 2011 (Table 3).

**Table 3:** Origin of blood meals obtained from *Culex pipiens pipiens* and *Culex restuans* in urban (Mercer County) and suburban (Monmouth County) habits during 2008–2011.

	Mercer County (% [95% CI])							Monmouth County (% [95% CI])					Total
	*Cx. p. pipiens*			*Cx. restuans*			Subtotal	*Cx. p. pipiens*		*Cx. restuans*		Subtotal	
Host Species	2009	2010	2011	2009	2010	2011		2008	2011	2008	2011		
American robin (*Turdus migratorius*)	1 (6.7 [0.1–19.3])	10 (32.3 [15.8–48.7])	4 (26.7 [4.3–49.1])			1 (50.0 [0.1–99.0])	16 (23.5 [13.5–33.6])	1 (11.1 [0.1–31.6])	26 (46.4 [33.4–59.5])	1 (20.0 [0.1–55.1])	10 (47.6 [26.3–69.0])	38 (41.8 [31.6–51.9])	54 (34.0 [26.6–41.3])
Northern cardinal (*Cardinalis cardinalis*)	2 (13.3 [0.1–30.5])	6 (19.4 [5.5–33.3])	1 (6.7 [0.1–19.3])			1 (50.0 [0.1–99.0])	10 (14.7 [6.3–23.1])	1 (11.1 [0.1–31.6])	5 (8.9 [1.5–16.4])	2 (40.0 [0.1–82.9])	1 (4.8 [0.1–13.9])	9 (9.9 [3.8–16.0])	19 (12.0 [6.9–17.0])
House finch (*Carpodacus mexicanus*)	4 (26.7 [4.3–49.1])	3 (9.7 [0.1–20.1])	2 (13.3 [0.1–30.5])				9 (13.2 [5.2–21.3])	3 (33.3 [2.5–64.1])	5 (8.9 [1.5–16.4])		1 (4.8 [0.1–13.9])	9 (9.9 [3.8–16.0])	18 (11.3 [6.4–16.3])
European starling (*Sturnus vulgaris*)	1 (6.7 [0.1–19.3])	2 (6.5 [0.1–15.1])	2 (13.3 [0.1–30.5])	2 (50.0 [1.0–99.0])	1 (100)		8 (11.8 [4.1–19.4])		5 (8.9 [1.5–16.4])		4 (19.1 [2.3–35.8])	9 (9.9 [3.8–16.0])	17 (10.7 [5.9–15.5])
Mourning Dove (*Zenaida macroura*)		1 (3.2 [0.1–9.5])	1 (6.7 [0.1–19.3])	2 (50.0 [1.0–99.0])			4 (5.9 [0.3–11.5])	2 (22.2 [0.1–49.4])	4 (7.1 [0.4–13.9])	1 (20.0 [0.1–55.1])	1 (4.8 [0.1–13.9])	8 (8.8 [3.0–14.6])	12 (7.6 [3.4–11.7])
House Sparrow (*Passer domesticus*)	3 (20.0 [0.1–40.2])	2 (6.5 [0.1–15.1])	2 (13.3 [0.1–30.5])				7 (10.3 [3.1–17.5])		2 (3.6 [0.1–8.4])	1 (20.0 [0.1–55.1])		3 (3.3 [0.1–7.0])	10 (6.3 [2.5–10.1])
American crow (*Corvus brachyrhynchos*)	2 (13.3 [0.1–30.5])		1 (6.7 [0.1–19.3])				3 (4.4 [0.1–9.3])		1 (1.8 [0.1–5.3])			1 (1.1 [0.1–3.2])	4 (2.5 [0.1–5.0])
Common grackle (*Quiscalus quiscula*)									3 (5.4 [0.1–11.3])		1 (4.8 [0.1–13.9])	4 (4.4 [0.2–8.6])	4 (2.5 [0.1–5.0])
Carolina chickadee (*Poecile carolinensis*)	1 (6.7 [0.1–19.3])	1 (3.2 [0.1–9.5])					2 (2.9 [0.1–7.0])	1 (11.1 [0.1–31.6])				1 (1.1 [0.1–3.2])	3 (1.9 [0.1–4.0])
Red-winged blackbird (*Agelaius phoeniceus*)									1 (1.8 [0.1–5.3])		1 (4.8 [0.1–13.9])	2 (2.2 [0.1–5.2])	2 (1.3 [0.1–3.0])
Song sparrow (*Melospiza melodia*)			1 (6.7 [0.1–19.3])				1 (1.5 [0.1–4.3])						1 (0.6 [0.1–1.9])
Rock dove (*Columba livia*)			1 (6.7 [0.1–19.3])				1 (1.5 [0.1–4.3])						1 (0.6 [0.1–1.9])
Northern oriole (*Icterus galbula*)									1 (1.8 [0.1–5.3])			1 (1.1 [0.1–3.2])	1 (0.6 [0.1–1.9])
Brown thrasher (*Toxostoma rufum*)								1 (11.1 [0.1–31.6])				1 (1.1 [0.1–3.2])	1 (0.6 [0.1–1.9])
Cedar waxwing (*Bombycilla cedrorum*)											1 (4.8 [0.1–13.9])	1 (1.1 [0.1–3.2])	1 (0.6 [0.1–1.9])
Yellow-crowned night heron (*Nyctanassa violacea*)											1 (4.8 [0.1–13.9])	1 (1.1 [0.1–3.2])	1 (0.6 [0.1–1.9])
Gray squirrel (*Sciurus carolinensis*)	1 (6.7 [0.1–19.3])	3 (9.7 [0.1–20.1])					4 (5.9 [0.3–11.5])						4 (2.5 [0.1–5.0])
Cat (*Felis catus*)		1 (3.2 [0.1–9.5])					1 (1.5 [0.1–4.3])		2 (3.6 [0.1–8.4])			2 (2.2 [0.1–5.2])	3 (1.9 [0.1–4.0])
Virginia opossum (*Didelphis virginiana*)		2 (6.5 [0.1–15.1])					2 (2.9 [0.1–7.0])		1 (1.8 [0.1–5.3])			1 (1.1 [0.1–3.2])	3 (1.9 [0.1–4.0])
Total no. identified (%)	15 (100)	31 (70.5)	15 (100)	4 (100)	1 (100)	2 (100)	68 (84.0)	9 (56.3)	56 (87.5)	5 (45.5)	21 (80.8)	91 (77.8)	159 (80.3)
Total no. tested	15	44	15	4	1	2	81	16	64	11	26	117	198

Percentages are provided in parentheses followed by ±95% CI.

*Culex* mosquitoes were predominately ornithophagic (n = 149, 93.71%) with only a small proportion feeding on mammalian hosts (n = 10, 6.29%) (Table 3). In Mercer County, the avian blood meal hosts of *Cx. p. pipiens* included 16 avian species (88.52%), while mammalian blood meals were obtained from only three species (11.48%). Mammalian blood was not detected in *Cx. restuans* from Mercer County, whereas avian blood meals were derived from four species (Table 3). In Monmouth County, avian hosts of *Cx. p. pipiens* included 12 species (95.39%), while mammalian blood meals were obtained from only two species (4.62%). No mammalian blood was detected in *Cx. restuans* from Monmouth County and avian-derived blood meals were obtained from ten species (Table 3).

## Discussion

Our investigations provide insight into the host associations of *Ae. albopictus* in the northernmost boundary of their established populations in eastern USA. Currently, about one-third of the human population of 55 million in this region reside in urban areas where *Ae. albopictus* is pervasive. This number is predicted to double under forthcoming climate change scenarios, encompassing all major urban centers and placing over 30 million people under the threat of dense *Ae. albopictus* infestations and potential public health threats from associated emerging mosquito-borne diseases [5]. Our analyses on the blood feeding behavior of *Ae. albopictus* demonstrate that this species is primarily mammalophagic in peridomestic environments of northeastern USA, and in some locations over 60% of their blood meals are derived from humans.

Host preference studies involving *Ae. albopictus* are often limited by the low sample numbers of blooded mosquitoes that are collected. This is because blooded *Ae. albopictus* have been difficult to collect [26], [32]. Previous sampling methods have often used combinations of aspirators, sweep nets, human baits, sticky traps, carbon dioxide-baited traps, and gravid traps in order to increase catch counts and as mentioned, often sampled exclusively in high density areas such as tire yards and dumps [17], [19], [20], [30], [31]. But trapping methods may bias results significantly [60], and *Ae. albopictus* is not readily attracted to traditional types of vector surveillance traps [26], [37]. A consistent sampling tool was not available for *Ae. albopictus* until the development of the BGS trap, which allowed us to sample populations of this species across a large geographic area over multiple years [42], [43]. Although we primarily utilized BGS traps for surveillance of host seeking *Ae. albopictus*, these traps also collected blooded specimens, which were subjected to molecular testing to characterize host feeding patterns of this species. However, unlike blooded or black blooded *Culex* mosquitoes which are easy to discern visually, blooded *Ae. albopictus* (unless fully engorged on fresh blood) are problematic to ascertain. This is because *Ae. albopictus* is a smaller species that imbibes smaller blood meals [18], [21] or on multiple hosts [61], [62], and contains a darker integument which hinders accurate detection of blood meals [32], particularly those in later Sella stages of development [26]. For example, parity studies conducted within our sampling sites on 166 *Ae. albopictus* visually determined as unengorged, detected blood meals or eggs in over 28% of those samples (Farajollahi et al. unpublished data). Our field investigations collected over 400 blooded *Ae. albopictus* during 2008–2011, 289 of which contained amplifiable blood for host determination analyses, with a successful amplification rate of close to 60%. In contrast, amplification rates were much higher for *Culex* mosquitoes (80%), likely because bird blood is nucleated and amplification of target DNA is easier for identification [53]. Interestingly, we collected twice as many blooded *Culex* mosquitoes than blooded *Ae. albopictus*, despite the demonstrable specificity of the BGS trap for the latter species. Amplification rates for *Ae. albopictus* also varied between the seasons and counties, as several abnormal weather patterns were experienced, threatening specimen handling and maintenance of the cold chain. The summers of 2010–2011 were particularly detrimental for blooded *Ae. albopictus* because the excessive heat (warmest and 3^rd^ warmest summers on record) may have desiccated specimens much faster in the BGS traps and reduced amplifiable DNA through degradation (http://climate.rutgers.edu/stateclim_v1/data). Nonetheless, successful blood meal results from 165 *Ae. albopictus* across a consistent spatial/temporal span provides valuable insight into the host associations of this species in the northeastern USA.

Our investigations are consistent with previous studies that have shown a high mammalian affinity by invasive *Ae. albopictus* in temperate areas of USA and Europe [19], [20], [25], [27], [31], [32]. However, unlike most of these studies, we did not document avian-derived blood meals in any of our *Ae. albopictus* samples despite extensive testing with avian-specific primers. Our findings cannot be attributed to the method of collection, blood meal identification methodology, host availability, or spatial/temporal factors, since the *Culex* mosquitoes collected in the same traps at the same time, were found to feed predominately on birds within our study sites as expected [59], [63], [64]. The lack of blood meals obtained from birds by *Ae. albopictus* suggest that this species may have limited exposure to endemic avian arboviruses, such as West Nile virus (WNV), which is supported by the lack of WNV isolations in over 34,500 specimens assayed in a complementary study [65]. However, the high mammalian affinity of *Ae. albopictus* suggests that this species may be an efficient vector of mammal-driven zoonoses such as La Crosse virus, and human-driven anthroponoses such as DENV and CHIKV.

Another concern regarding the vectorial capacity of *Ae. albopictus* stems from detection of multiple blood meals from field populations. Previous studies have documented vertebrate blood from more than one host in *Ae. albopictus* throughout its endemic and invasive range (Table 1). Our studies detected double blood meals in 6% of the field-collected *Ae. albopictus* specimens, consistent with the 6% to 10% double blood meal proportion rates reported by others [22], [26], [27], [30], [31]. The capacity for *Ae. albopictus* to acquire multiple blood meals, particularly from human and other host species, increases the vector potential of this mosquito because of greater exposure to infected hosts during multiple feedings.

Large proportions of human-derived blood meals have been documented previously in *Ae. albopictus* and a few studies have reported that field populations feed exclusively on humans (Table 1), but the use of aspirators and human bait may bias these estimates. Additionally, recent investigations in temperate Italy have shown that *Ae. albopictus* feeding patterns differ between urban and rural habitats, with 90% of blood meals in urban areas from humans and only 20% being human-derived in rural habitats [31]. Our results report a significantly higher proportion of human blood meals in *Ae. albopictus* from suburban areas, rather than the densely populated urban areas. This was surprising, because of the higher (>2 times) human population density in urban Mercer County. However, suburban dwellers often spend more time outdoors gardening or undertaking leisure activities in backyards during daylight hours which will increase exposure. In addition, proportions of *Ae. albopictus* feeding on cats and dogs was higher in urban than suburban sites, likely reflecting large populations of feral cats in urban low income areas [66] and the fact that often dogs are kept in outside cages or yards for homeowner protection [40]. In contrast, suburban residents primarily keep their pets indoors and availability of these hosts for *Ae. albopictus* may be reduced. The significantly greater anthropophagic behavior of *Ae. albopictus* in more affluent suburban versus low-income urban habitats of northeastern USAindicates that a larger public health concern may exist within suburban landscapes, despite lower human population densities. Higher proportions of *Ae. albopictus* feeding on cats and dogs within urban environs may help fuel local mosquito populations but it may also afford zooprophylaxis protection for humans during epidemic outbreaks of anthroponoses such as DENV or CHIKV, because it will divert vector feeding to non-susceptible dead-end hosts.

### Summary and Public Health Implications

Recent decades have witnessed a dramatic global expansion of *Ae. albopictus* into temperate areas and an increase in locally acquired autochthonous cases of tropical diseases such as DENV and CHIKV [9], [11], [67]. Because of the increasing abundance of *Ae. albopictus* and the escalating diagnoses of exotic pathogens in travelers returning from endemic or epidemic areas [14], the risk of a tropical disease outbreak in a new area is no longer speculative. We have shown that in urban and suburban areas of temperate northeastern USA, invasive populations of *Ae. albopictus* fed exclusively on mammalian hosts and that a large proportion (50–60%) fed on human hosts. Although we did not detect any avian-derived blood meals from *Ae. albopictus* during our investigations, the species has been traditionally classified as an opportunistic feeder whose host preference is greatly dependent on the abundance of available local hosts [18], [21]. Our studies indicate that *Ae. albopictus* may play a greater role in anthroponoses disease cycles, such as DENV and CHIKV, and a lesser role in zoonoses involving an avian animal reservoir. However, we cannot rule out the possibility that *Ae. albopictus* may occasionally act as a bridge vector for endemic pathogens such as St. Louis encephalitis virus and WNV by feeding on infected hosts when their abundance is great. Nonetheless, the large and growing populations of *Ae. albopictus* in major metropolitan urban and suburban centers, make a large autochthonous outbreak of an arbovirus such as CHIKV or DENV a clear and present danger. This may be particularly imminent in the case of CHIKV, as the virus is explosively spreading in the Caribbean region of the western hemisphere for the first time [68]. Given the difficulty in successful suppression of *Ae. albopictus* in areas where it has become firmly established [5], [43], we strongly recommend further ecological investigations on this species and caution public health practitioners and policy makers to install proactive measures for the imminent mitigation of an exotic pathogen outbreak.

## Supporting Information

Table S1 Number of *Aedes albopictus* collected by BGS traps in Mercer and Monmouth Counties during 2008–2011.
(XLSX)

Table S2 Number of *Culex pipiens pipiens* and *Culex restuans* mosquitoes collected by BGS traps in Mercer and Monmouth Counties during 2008–2011. Some specimens were not morphologically identified to species and were enumerated as *Culex* spp. U = unengorged, BF = blood fed, BB = black blooded, G = gravid.
(XLSX)
